# OMICS approaches in cardiovascular diseases: a mini review

**DOI:** 10.5808/gi.21002

**Published:** 2021-06-30

**Authors:** Md. Mehadi Hasan Sohag, Saleh Muhammed Raqib, Syaefudin Ali Akhmad

**Affiliations:** 1 Department of Genetic Engineering and Biotechnology, Jagannath University, Dhaka 1100, Bangladesh; 2 Biotechnology Research Initiative for Sustainable Development, Dhaka 1219, Bangladesh; 3 Baridhara Scholar’s Institution, Dhaka 1212, Bangladesh; 4 Department of Biochemistry, Faculty of Medicine, Islamic University of Indonesia, Yogyakarta 55584, Indonesia

**Keywords:** cardiovascular diseases, genomics, metabolomics, OMICS, proteomics, transcriptomics

## Abstract

Ranked in the topmost position among the deadliest diseases in the world, cardiovascular diseases (CVDs) are a global burden with alterations in heart and blood vessels. Early diagnostics and prognostics could be the best possible solution in CVD management. OMICS (genomics, proteomics, transcriptomics, and metabolomics) approaches which could be able to tackle the challenges against CVDs. Genome-wide association studies along with next-generation sequencing with various computational biology tools could lead a new sight in early detection and possible therapeutics of CVDs. Human cardiac proteins are also characterized by mass spectrophotometry could open the scope of proteomics approaches in CVD. Besides this, regulation of gene expression by transcriptomics approaches exhibits a new insight while metabolomics is the endpoint on the downstream of multi-omics approaches to confront CVDs from the early onset. Although a lot of challenges needed to overcome in CVD management, OMICS approaches are certainly a new prospect.

## Introduction

In this 21st century, cardiovascular diseases (CVDs) ranked as one of the serious health issues of the developed and developing countries that encompass so many factors such as tissue, organs and multidimensional molecular perturbations make CVD a global burden [1]. World Health Organization declares CVD as the topmost cause of death throughout the world. In 2015, 17.9 million people died from CVDs which represents 31% of all diseases where cardiac arrest is one of the biggest portions of CVDs. Global Heart Atlas affirmed that 200 million prevalent peripheral artery disease cases were estimated in the last decade where three fourth of the population are living in low or middle-income countries, specifically most of the patients are from the southern region of Asia [2]. In the era of the coronavirus disease 2019 (COVID-19) pandemic, CVD becomes comorbid that increased the risk of getting higher morbidity and mortality of COVID-19 [3]. British Heart Foundation defines CVD as a total of all the diseases of the heart and circulation including coronary heart diseases, angina, heart attack, congenital heart diseases, and stroke [4]. As various researchers describe that CVD is enormously influenced by differences in environmental parameters and lifestyle attributes while socioeconomic conditions causing metabolic diseases and age risk factors are also playing critical roles other than because of infection [5,6]. Besides this, the genetic makeup of the individual is one of the pivotal factors of CVD prognostics and diagnostics where OMICS technology could be recommended for further investigations [7]. OMICS is nothing but promising techniques where the combination of genomics, transcriptomics, proteomics, and metabolomics are conglomerated into an umbrella where researchers can predict the probable identification of the disease and make the best solution for the remedy [8].

## Clinical Spectrum of CVD and Its Laboratory Testing Biomarker

CVD has various symptoms and manifestations as well as its laboratory testing biomarkers. Common signs and symptoms of CVD are associated with a heart problem and vascular problem attributed to which organ affected such as paresis when brain getting stroke disease. Other symptoms are chest pain, pain in the neck, jaw, throat back, and upper abdominal, fatigue, edema, dyspnea/shortness of breath, irregular heartbeat, nausea, loss of appetite, dizziness, numbness, and cyanosis, as well as easily tiring during exercise [9].

**Fig. 1.**
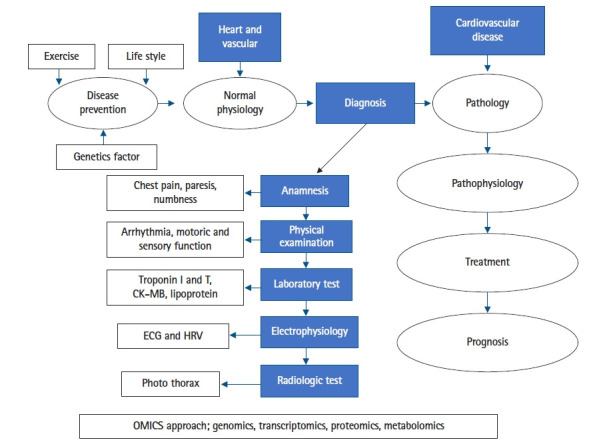
OMICS approach from diagnosis process until prognosis as well as the etiology of the disease. CK-MB, creatine kinase-myocardial band; ECG, electrocardiogram; HRV, heart rate variability.

CVD may have various clinical spectrum and various laboratory results as a biomarker profile in the diagnosis process, therapeutic monitoring, a predictor for prognosis as well as the etiology of the disease (Fig. 1). For example in the COVID-19 pandemic with a highly clinical spectrum of signs and symptoms of infection by severe acute respiratory syndrome coronavirus 2 (novel coronavirus) presented on a patient includes CVD manifestation affected both heart and vascular on all system. In terms of physiology, regarding the COVID-19 on clinical presentation with the various sign and symptom, there is a specific area of physiology having a pivotal role in exploring the cause-effect relationship and completing the biomarker profiles of the patient that is electrophysiology i.e., electrocardiogram and channel recording to uncover the pathophysiology of CVD. CVD phenotype with complete clinical presentation supported by electrophysiology will be beneficial to link CVD biomarker in terms of OMICS approaches supporting the signs as well as the symptoms of CVD patient. Table 1 depicts the biomarker of CVDs particularly in COVID-19 in comparison with other causes [10].

From the basic science perspective, that worthwhile to use OMICS approaches for mitigating that the argument of genetic differences may be at the core of differential disease expression in the sign and symptoms which may be integral to altered responses to standard medical therapies [11].

## OMICS Approaches for CVD Mitigation

**Fig. 2.**
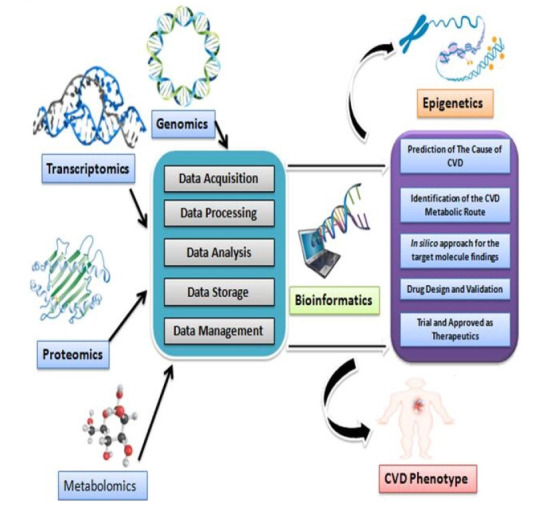
Graphical representation of OMICS workflow for cardiovascular diseases (CVDs) research. Idea of the picture is adopted from the literature review.

OMICS is an amalgamation of technologies which accumulates initially the universal detection of genes referred to as genomics, processing with proteins and peptides that termed as proteomics, analyzing with RNAs (e.g., mRNA) that defined as transcriptomics, and the study of metabolic molecules as metabolomics in a perspective of biological phenomenon. Additionally, next-generation sequencing (NGS), genome-wide association study (GWAS), RNA sequencing (RNA-Seq), mass spectrophotometry (MS), and other high throughput techniques are the blessing outcomes from the broad ranges of application of OMICS [12]. Data that are found from the OMICS are relevant and referred to *in silico* manners that are strictly standardized as computational approaches whereas it is said that OMICS records are explored into bioinformatics. Data sets are processed uniquely along with the procedures e.g., acquisition of data, processing and analysis, and storage. Nevertheless, the most influential part of the data processing system is nothing but the management of data which could be treated as big data management as a lot of sequences of nucleic acids from various organisms (genomic, plasmid, and so on) are needed to be retrieved frequently for multi-research purposes. Besides this, stored data are also needed to be managed for the epigenetic study which will play a key role to answer the mystery of life [13]. However, ‘OMICS’ approaches could lead the scientific community in identifying the metabolic pathway and responsible genes associated with CVDs which is the ultimate window opening scenario to design and validate the drugs that will undergo a clinical trial. A figurative outline based on a previous study has been drawn (Fig. 2) to express the general idea of OMICS workflow [14].

## Genomics in CVDs

Genomics approaches are esteemed as one of the most accurate strategies for the diagnostics and prognostics of CVDs. Single nucleotide polymorphisms (SNPs) are an example of the genomics technology that would be an imperative tool for CVD researches by identifying the candidate gene through the GWAS study. Already, there is stringent evidence that has confirmed in favor of SNPs that could identify biomarkers for various common diseases [15]. Besides this, genomics-related risk profiling and quantitative clinical measurements give a pathway for precision therapies for various cardiovascular abnormalities. In recent days, Genomic variation and phenotypic illness or wellness might be an indicator of clinical implication by Electronic Health Records and Genomics programs [16]. These programs are smoothly run by using clinical data from electronic medical records that is linked to various clinical repositories or biobanks including studies from eMERGE (Electronic Medical Records and Genomics; http://www.gwas.net) network and as well as Clinical Implementation of Personalized Medicine through Electronic Health Records and Genomics (CLIPMERGE PGx) [17]. Additionally, the National Institute of Health has taken an initiative through National Centre for Biomedical Computing (NCBC) entitled “Informatics for Integrating Biology and the Bedside (i2b2; https://www.i2b2.org)” where researchers have the user-friendly software analysis tools for collection and management of clinical data based on genomics research [18]. Besides this, hypertrophic cardiomyopathy was the inception case of the cardiovascular genetic disorder, where a missense mutation occurred in *β* myosin heavy chain was detected [19]. Mostly, the genome sequencing approaches came with great hope towards the scientific community for identifying DNA sequence variants (DSVs) which is the ultimate consequence of single gene abnormalities. NGS took the definitive place for revealing the gene defect that happened due to cardiovascular problems where all the DSVs are profoundly identified through whole-exome sequencing or whole-genome sequencing methods. Millions of DNA fragments are sequenced here in NGS, where phenotypic expression is also determined that allows investigators to draw out a clear picture of the disorder [20]. Already a report has been shown that intermediate polymorphism of several genes like angiotensin I converting enzyme, Chymase, Coagulation factor III and V have keeping up the adverse preoperative cardiovascular outcomes. Due to the changes in enzymatic activity and down-regulation of the receptors, polymorphism of the above genes can turn into an adverse cardiac event after bypass grafting [21]. Additionally, up-regulation of the Interleukin-6 level also put an unfavorable cardiac status after surgery. Another example of preoperative genomics is associated with platelet glycoprotein IIIa gene (ITGB3) polymorphism. This initiates the aggregation of platelet and increased the level of troponin1 which occur unpleasant results of a patient with surgery like coronary graft occlusion, myocardial infarction, and even death [22]. However, additional functional genomics studies of atrial-natriuretic peptide and brain natriuretic peptide denotes this molecule as biomarkers of heart failure, where the prior knowledge from whole genome picture minimizes the risks of the sudden demise of the patient [23].

## Proteomics in CVDs

Prior knowledge of protein function and characterization would be the promising solution to identify and mitigate the risk factors towards CVDs. As we know, proteomics approaches could demonstrate the protein patterns and as well as post-translational modifications that help to understand the genetic consequences of the final product of responsible gene which is not deeply predictable through the bioinformatics analysis and not also be confined by the genomics studies [24]. By achieving the genomic information, proteomics studies based on accumulating macromolecule databases with the advent of MS or Edman degradation would be done for further progress. The targeted proteome is extremely compared with the entire set of the known proteome to unveil the function and characteristics of disease-specific protein that is named functional proteomics [25]. However, plenty of sophisticated molecular biology research tools like resolving from a gel, electrospray ionization, matrix-assisted laser desorption/ionization are needed to prepare and ionize the sample while peptide sequencing, as well as peptide mass fingerprinting, make the mass spectrometric analysis, is so inevitable techniques in proteomics technology [26]. Though it is not completely understood about cardiac dysfunction in systemic as well as specific heart muscle diseases, considerable changes in myocardial gene and expression of proteins could be the determiner of CVD status. Additionally, two-dimensional electrophoresis was done as a proteomics tool to make a profound dataset of cardiac proteins of humans. Information of hundreds of proteins related to CVDs is deposited here for further analysis and comparison of newly found proteins [27]. Moreover, databases of human cardiac proteins along with the databases for other animals are used for constructing an animal model of heart diseases. Additionally, various reports confirmed that the identification and expression of the protein (e.g., genetic elements, toxic substances, antibodies, and so on) has already been investigated on dilated cardiomyopathy that gives new hope in CVD research [28]. Surprisingly, proteomics also put a positive vibe on cardiac transplantation where identification of cardiac-specific antigen identification might be the global approach. Besides these approaches, novel cardiac biomarkers are also identified through proteomics investigations. Previous studies have been clarified that levels of heat shock protein-27 in tissue samples are determiners of acute myocardial infarction (AMI). As a consequence, proteolytic factors in the blood are also treated as the new insight of biomarker identification strategies to early-stage detection of the acute coronary syndrome [29]. Another biomarker, gamma glutamyl transferase is also associated with the prediction of survival rate in patients with acute ischemic stroke [30]. It has been already well-known that about 177 candidate biomarker protein was reported where proteomics approaches could quantify the measurement of the target proteins [31]. Identification of the protein biomarker from blood serum or plasma is still challenging though a complementary proteomics strategy with multiplex immune analysis could validate the target biomarker. Surprisingly, collected fractionations of plasma/serum of relevant protein or protein complex named interactomes are characterized by immunocapture and liquid chromatography-mass spectrometry. Additionally, glycosylated protein biomarker like erythropoietin is another potential regulator in cardiac patient management [32]. Furthermore, C-reactive protein (CRP) is also regarded as one of the promising biomarkers to identify systemic inflammation in atherogenesis which shows a very close association with chemokines and cytokines for disease development. A molecular entity of the CRP proteins is also evaluated and quantified by the MS spectra [33].

## Transcriptomics in CVDs

As with other approaches, transcriptomics also plays an inevitable input in current days CVD research. As we know, that the expression of a gene is entirely written on the RNA as a transcript, so the techniques in transcriptomics made an easy pathway to detect the defective genes regarding CVDs in a very early stage. These techniques are efficient to identify as well as quantify all RNA transcripts that may be regulatory or not, as it is affirmed that occurrences of CVDs are dependent on activation or silencing of responsible cardiac genes. Usually, microarray and RNA-Seq are primary schemes that are used in this technology, where the second one is more comprehensive, specific, and costly than the previous one. Microarray denotes the quantification of RNA is somewhat similar to a genome-wide DNA microarray, while RNA-Seq is a high throughput technology is likely as NGS approaches of complementary DNA. To sum up the transcriptomic analysis, few protocols are used for the annotation of the gene expression, e.g., Heat maps, gene co-expression analysis network, pathway analysis, and so on. Above processing in the experiment would be capable of making a crystal clear visualization with a firm understanding of the entire transcript [34]. However, transcriptomics is the only option to reveal the impact of a variety of RNA e.g., micro RNA (miRNA), small interfering RNA (siRNA), non-coding RNA (ncRNA), and others, as they are thought as eminent players of the cell to cell communication and thought to be as a potential biomarker of CVD prognostics and diagnostics. The availability and stability of ncRNA are profoundly higher than the coding counterpart that could make themselves as intense therapeutic targets as they are not degraded in blood. Previous studies also affirm that the role of ncRNA in CVDs is tremendous because they are associated with different cardiovascular processes including heart development; fibrosis, heart contractility, apoptosis, gene regulation, and so on. This phenomenon will open the horizon of translational research gateway that could introduce personalized medicine in CVD management. Furthermore, quantitative real-time polymerase chain reaction (qPCR) with high throughput arrangement of computational biology gives a rigid strength to the microarray where RNA-Seq approaches give the entire information of complete RNA profiling. These inclusive approaches of transcriptomics disclose the way of identification, progression, and treatment of CVDs [35]. However, a recent study gives a picture of bioinformatics approaches through NGS platforms used in transcriptomics. By sequence mapping and quantifying the expression, RNA-Seq datasets represent the differential detection processes of expressed cardiac genes [36]. Circulating miRNA is reported as the signature molecule to identify the risk level of heart failure patients as well as AMI. Specifically, miRNA-21, miRNA-208, miRNA-423, and miRNA-49 are responsible for heart failure and myocardial infarction, while miRNA-1, miRNA-133, miRNA-328 have also been identified as a biomarker of arrhythmia. Besides this, miRNA-22 could be treated as therapeutics against cardiac autophagy. Overexpression of miRNA-99a in animal models proved that it could improve cardiac function [37]. Samples from the patients with adverse cardiac events could easily be distinguished by the estimation of miRNAs through real-time qPCR due to the cardiac selectivity and plasma stability with rapid release of these biomarkers [38].

## Metabolomics in CVDs

Disturbances in metabolic signals alter the usual characteristics of the organ system and the cardiovascular system is not an exception. Contemporarily, the notion of CVDs could alter the fates of the intermediate metabolites in different metabolic pathways, which signs a new thought on the pathophysiological status of a CVD patient. In terms of CVD, there is evidence that because lack of endogenous ascorbate leads to the underlying cause of CVD compared with the animal having higher endogenous ascorbate [39]. Alongside other technologies, metabolomics will also offer insight into the diagnosis and prognosis of CVDs through the prediction and analysis of cellular metabolism [14]. Reports illustrate that metabolomics stands on the endpoint of the downstream circle of OMICS approaches where biological fluids or tissue samples are analyzed with nuclear magnetic resonance (NMR) and liquid chromatography-based mass spectrometry (LC/MS) as well. In terms of specificity and throughput attribute, NMR is superlative to targeted and non-targeted LC/MS while the opposite scenario is observed in the case of sensitivity and metabolome coverage [40]. Furthermore, analysis in ketogenic and glucogenic regulation alongside macromolecular metabolic pathways, metabolome like circulating branched-chain amino acids could interact with insulin resistance results from the prognosis and better treatment of CVDs with comorbidities like obesity and diabetes. Besides this, lipid metabolism alteration is also regarded as one of the strongest incidents for CVD occurrences. Metabolomics study could unveil the early detection of specific cholesterol esters, choline, triacylglycerols, and a density level of lipoproteins considerably associated with chronic heart disease. Additionally, metabolomics strategies also describing inborn metabolic errors which could be associated with thousands of metabolites in cardiometabolic pathways [14,40]. Metabolic screening of choline, trimethylamine N-oxide, and betaine leads towards a conclusion of the association of dietary metabolites with the development of CVDs. Hence, persistent arterial fibrillation is also regarded as the hypermetabolic state where glucose metabolism is impaired and prominently associated with ketone bodies [41]. Recent updates in metabolomics study depict the early vascular aging syndrome motives which are firmly associated with cardiovascular morbidity and mortality. Arterial stiffness is measured by four lysophosphatidylcholines that predict the risks of hypertension as well [42]. Till now, more than eight thousand metabolomes are enlisted into databases of human metabolome projects that actively take part in the understanding of system biology. Already biomarkers of myocardial ischemia are known where approximately six metabolites are confirmed. Another recent study has stated that eighteen dietary metabolites could signal the early onset [40,41]. Reprogrammed cardiac metabolic pathway increased the uptake of glucose due to cardiac hypertrophy where fatty acid oxidation decreased to a minimum level. This metabolic alteration leads the patients towards heart failure. Besides this, a considerable amount of changes are found in fatty acid oxidation in the time of transition to heart failure. Here, LC-MS-based metabolomics showed a decreased level of TCA cycle intermediates [43].

Novel metabolic pathways related to an inflammatory protein like fibrinogen and CRP are not confined yet irrespective of the age, gender, diet, or drug effects of the patients. However, MS-dependent metabolomics approaches initiate the eye-catching processes of detection based on physiological attributes in targeted or untargeted metabolic pathways for assessing the risks of CVDs with possible remedy recommendations [44].

## Conclusion

Numerous numbers of researchers are engaging in CVD researches to find out the way of an ultimate solution against this global burden. It is certain that, for the improvement of CVD management, early diagnosis is a must. Multi-omics approaches have opened a broad avenue to detect the early onset of CVDs symptom though a long way to go for the inclusive conclusion. However, the Integration of OMICS strategies with clinical researches will be a strong shield to fight against CVDs.

## Figures and Tables

**Table 1.** The biomarker of CVDs particularly in COVID-19 [10]

Cardiac biomarker	Definition	Association with COVID-19	Prognostic potential
cTn	Gold-standard necrotic biomarkers for myocardial injury	Acute myocardial injury, ICU admission, in hospital death and severity of inflammation in COVID-19	+++
BNP	Predictor of adverse outcome following acute myocardial injury	Acute myocardial injury, ICU admission, in hospital death	++
CK-MB	Biomarker of myocardial damage and reperfusion	Acute myocardial injury, ICU admission, in hospital death	+

CVD, cardiovascular disease; COVID-19, coronavirus disease 2019; cTn, cardiac troponin; ICU, intensive care unit; BNP, brain natriuretic peptide; CK-MB, creatine kinase-myocardial band.
